# Coronary‐cameral fistula in an infant patient: An incidental diagnosis

**DOI:** 10.1002/ccr3.8172

**Published:** 2023-11-17

**Authors:** Raúl Velázquez‐Castañeda, Ilana De La Puente‐Tawil, Sofía Molina‐Castillo, Leonardo Proaño‐Bernal, Mariell Alejandra Rodríguez‐Salazar, Steve Masso‐Bueso, Erick Alexanderson‐Rosas, Vincenzo Arenas‐Fabbri

**Affiliations:** ^1^ Instituto Nacional de Cardiología Ignacio Chávez Mexico City Mexico; ^2^ Universidad Nacional Autónoma de Mexico Mexico City Mexico; ^3^ Universidad Anahuac Mexico Mexico City Mexico

**Keywords:** cardiac catheterization, computed tomography angiography, congenital heart defects, coronary artery anomalies, coronary‐cameral fistula

## Abstract

Coronary artery fistula is a rare abnormal connection between the heart arteries. Patients may remain asymptomatic until adulthood, potentially experiencing life‐threatening complications. Adequate monitoring and therapeutic management are essential.

## Graphical abstract

Coronary artery fistula is a rare abnormal connection between the heart arteries. In this clinical case, we present a 3‐month‐old female who had an intercoronary communication between the left anterior descending artery and the right coronary artery in the mid‐segment, both of which were tortuous and dilated. Patients may remain asymptomatic until adulthood, potentially experiencing life‐threatening complications such as aneurysm formation, intimal ulceration, calcification, side‐branch obstruction, mural thrombosis, and rupture. Adequate monitoring and therapeutic management are essential.

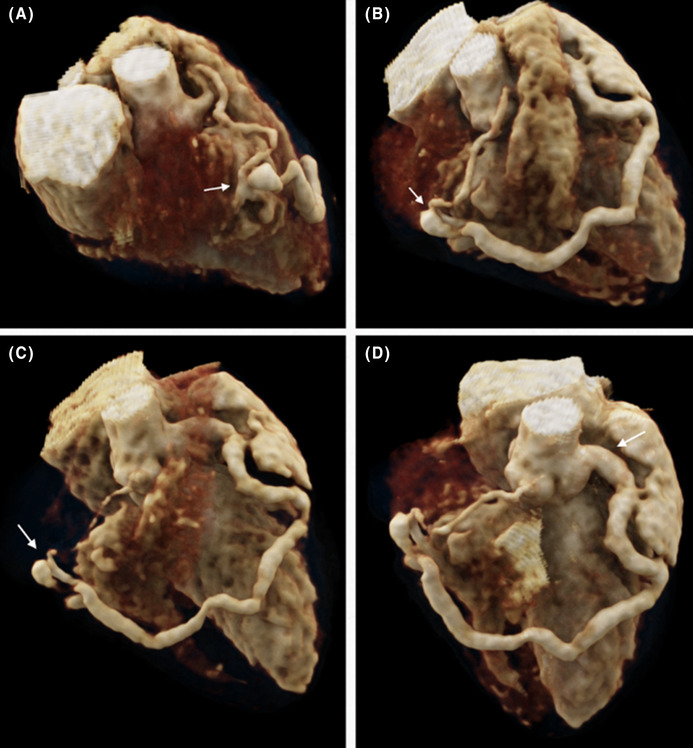

## 1 INTRODUCTION

Coronary artery fistulas (CAFs) can be defined as an uncommon abnormal connection between coronary arteries and either heart chambers or major thoracic vessels, allowing blood to bypass the usual capillary network in the myocardium. If these connections terminate in heart chambers, they are termed coronary‐cameral fistulas, whereas those ending in veins are referred to as coronary arteriovenous fistulas. Congenital forms of CAF are more frequent, but still only make up just 0.4% of all congenital cardiac abnormalities.^1^ The majority of patients have a solitary CAF; however, approximately 20% of patients exhibit fistulas originating from two or more coronary arteries.^2^

The clinical presentation of CAFs varies according to factors such as the patient's age, flow volume, the recipient chamber's resistance, and the development of myocardial ischemia. Often, the anomaly is inadvertently discovered during routine examinations or coronary angiography, with the condition being recognized due to the presence of a continuous murmur upon examination.^3^, ^4^

Compared to other imaging modalities, computed tomography angiography (CTA) is highly valuable for the assessment of CAFs due to a shorter acquisition time and the ability to provide superior temporal and spatial resolution. Multiplanar reconstruction along with 3D volume‐rendered imaging offers exceptional anatomical details, encompassing the origin, trajectory, and drainage location of CAFs. This holds true even for intricate anomalies, thereby establishing its potential as an essential tool for guiding treatment planning.^5^

Regarding treatment, surgical or catheter‐based closure is highly recommended for symptomatic patients and asymptomatic patients with high‐flow shunting, particularly in pediatric cases. Interventional closure has become the preferred approach, especially in younger patients, although the method choice also considers fistula anatomy, the presence of other cardiac defects, and the expertise of the interventional cardiologist.^6^

## 2 CASE PRESENTATION

A 7‐day‐old female patient presented at a local clinic for newborn metabolic screening. The patient displayed symptoms of dehydration and jaundice, along with an unspecified heart murmur. The mother reported substance abuse (alcohol, cannabis, and cocaine) during pregnancy. After birth, the patient was hospitalized for 7 days due to hyperbilirubinemia, microcephaly, and an unspecified congenital heart defect.

After consulting a pediatric cardiologist, a right ventricular‐coronary fistula diagnosis was suspected. Treatment with furosemide and spironolactone was initiated, and the patient was referred to a third‐level hospital at 3 months of age with a height of 0.52 m and a weight of 5 kg, for further assessment and management. During the examination, the patient appeared stable and asymptomatic. Upon auscultation, a regurgitant murmur at the mesocardium was identified, with no other significant findings.

## 3 DIFFERENTIAL DIAGNOSIS, INVESTIGATIONS, AND TREATMENT

**FIGURE 1:**
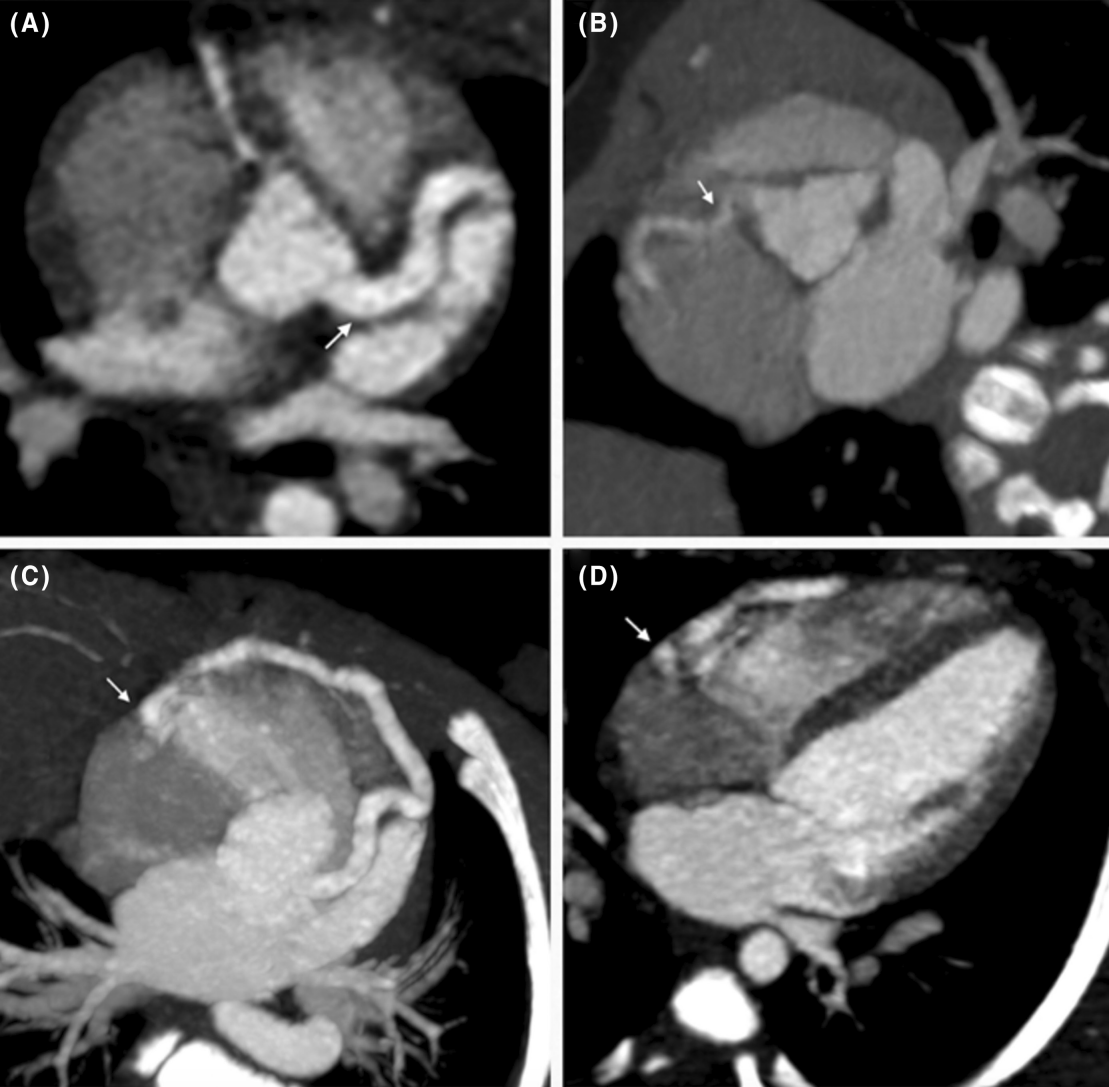
Coronary CT angiography findings: (A) ectasia of the left main coronary artery (4.7 mm, Z 10.9) and (C) proximal segment of the left anterior descending artery (3.9 mm, Z 10.2), where it forms a fistula that crosses the interventricular septum and the right ventricle (RV) free wall (arrow). (B) Proximal normal‐sized right coronary artery. (D) Four‐chamber view showing the coronary fistula drainage into the basal third RV free wall, a discernible opacification difference between the right cavities is also appreciated.

**FIGURE 2:**
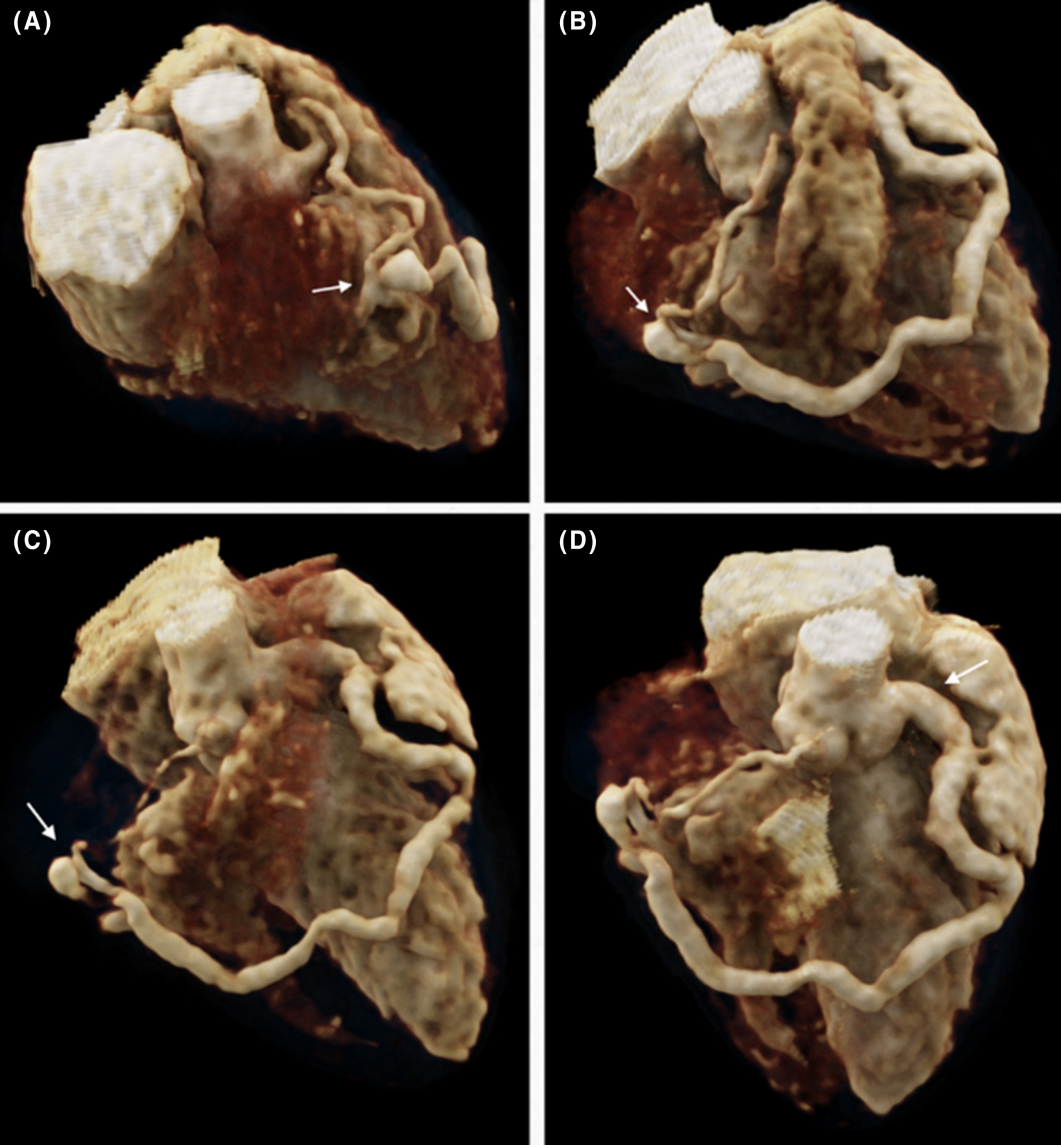
Coronary CT angiography volume render reveals the following observations: (A) right coronary artery (RCA) proximal tortuosity and midsegment dilation, where it joins the left anterior descending artery (LAD) fistula prior to its drainage into the right ventricle (RV). (B, C) The “arched” trajectory of the LAD coronary fistula and its connection with the RCA is evident, with its entry point situated at the basal third of the RV free wall. (D) Dilation of the left main coronary artery and the LAD, abnormal path of the LAD up to its convergence with the RCA.

The decision was made to proceed with a transthoracic echocardiography (TTE) and a CTA. The CTA revealed an intercoronary communication between the left anterior descending artery and the right coronary artery in the midsegment, both of which were tortuous and dilated (Figures 1 and 2). They present an “arched” morphology by merging before draining into the basal third of the free wall of the right ventricle (RV). Additionally, there was coronary ectasia in the left main coronary artery.

A multidisciplinary team, including the hemodynamics department, decided for conservative management and a 6‐month follow‐up was scheduled along with a TTE evaluation, it was explained to the mother that if any symptoms occur (dyspnea, fatigue), she has an open appointment at our hospital. To date, there have been no such symptoms reported.

## 4 DISCUSSION

Congenital CAFs occur with equal frequency in both sexes, there is no research to suggest a correlation to ethnicity. In the general population, the occurrence of coronary anomalies ranges from 0.2% to 1.2%, with CAFs constituting 0.002% of these cases. Among CAFs, solitary fistulas are more prevalent, and approximately 75% of incidentally discovered CAFs exhibit no clinical symptoms.^1^

The RV serves as the most common drainage site for CAFs, representing 34.9% of reported cases. The right atrium and the pulmonary artery rank as the second most frequent drainage sites, each comprising 27% of cases, followed by the left ventricle at 6.3%. The coronary sinus accounts for 3.2% of cases, and lastly, the left atrium makes up 1.6% of occurrences.^7^

The most common types of CAFs have a congenital etiology. Anomalies in the coronary arteries can arise from various sources, including the persistence of rudimentary embryonic coronary arterial structures, disruptions in normal development or atrophy processes, and misplacement of connections within an otherwise typical coronary artery.^1^, ^8^

CAFs can be classified based on their size: small, medium, or large, depending on whether the fistula diameter is <1, between 1 and 2, or >2 times the largest diameter of the coronary vessel that does not supply the coronary fistula. They can also be categorized according to the drainage site: coronary‐cameral fistula (the most common), coronary‐to‐pulmonary artery fistula, coronary artery‐to‐coronary sinus, and coronary artery‐to‐bronchial artery fistula.^2^, ^9^ A shunt from left to right (coronary artery to right vessels or chambers) leads to an ongoing flow throughout the entire cardiac cycle, driven by the lower pressure within the right structure (vessel or chamber) in comparison to the myocardial capillaries or arterioles. This often gives rise to a volume overload on the right side, although this pathophysiological mechanism can also involve chambers on the left side.^1^

Symptoms typically arise around age 18, with dyspnea being the primary symptom, accompanied by fatigue, congestive heart failure, and pulmonary hypertension. Possible complications encompass coronary artery dilation, aneurysm formation, intimal ulceration, medial degeneration, intimal rupture, atherosclerotic deposition, calcification, side‐branch obstruction, mural thrombosis, and rupture. Notably, angina pectoris is rare without arteriosclerotic coronary artery disease.^3^, ^4^

A characteristic physical finding in patients with CAFs is the presence of a gentle, continuous murmur. This murmur typically follows a crescendo–decrescendo pattern in both systole and diastole, with its intensity being more pronounced during diastole.^3^ Depending on the location where the fistula connects to the heart, the murmur will be most audible at certain points on the chest wall.^4^

Diagnosing CAFs can be challenging. Initial assessment includes an electrocardiogram, with findings based on the fistula's location and flow. Selective invasive coronary angiography used to be the reference standard. It enables precise visualization of the anatomy of the CAF, including fine vessels, with high temporal and spatial resolution and yields hemodynamic information. In addition, it facilitates the diagnosis and therapeutic embolization. However, conventional coronary angiography is invasive and involves the risk of procedure‐related complications. Furthermore, it yields two‐dimensional projection images, which are often limited in the delineation of the complex anatomy of abnormal communications, with reported correct diagnosis rates of 35%–50%.^5^

Selective invasive coronary angiography has been replaced by TTE, the now preferred initial assessment method. TTE can show dilated coronary arteries (CAFs that are large and/or have a diameter greater than 3 mm) and distal drainage via color flow mapping in CAF cases.^9^ However, it is less effective for small shunts and pulmonary artery fistulas. Microbubbles enhance color Doppler signals to pinpoint CAF locations.^7^ Two‐dimensional echocardiography displays heart enlargement and function, but not fistula function.^4^

Multidetector computed tomography is a valuable alternative to echocardiography and catheter angiography for evaluating anomalies, with its increased use leading to enhanced anomaly recognition due to improved sensitivity in volumetric data acquisition and preprocedural planning for patients with larger communications to specific heart chambers, defining fistula characteristics and assisting in treatment approach decisions, device selection, embolization predictions, and optimal fluoroscopic angle identification.^7^, ^10^

Contemporary dual‐source computed tomography (DSCT) with ECG gating provides high‐resolution images in a shorter time frame through a single breath‐hold, with superior temporal and spatial resolution compared to magnetic resonance imaging. Volume‐rendered images from three‐dimensional computed tomography (CT) data sets offer comprehensive views of cardiac and vascular anatomy, aiding surgical planning by clarifying anatomical complexities. The primary downside of CT lies in radiation exposure risk, which can be mitigated by modern DSCT scanners and advanced dose reduction techniques.^7^, ^10^

The updated 2018 American College of Cardiology/American Heart Association guidelines underscore the significance of a collaborative heart team approach to assess the suitability and feasibility of CAFs closure at centers proficient in both percutaneous and surgical closure techniques.^11^

Common clinical scenarios warranting consideration for CAFs closure encompass evidence of ischemia in the feeder artery territory, arrhythmias suspected to be linked to CAFs, endarteritis, vessel rupture, cardiac chamber enlargement, and ventricular dysfunction. It is crucial to highlight that small CAFs tend to close spontaneously over time, allowing for monitoring without intervention. In contrast, medium‐ to large‐sized fistulas can expand, particularly in pediatric and young adult patients, often associated with proximal coronary artery dilation signifying prolonged high‐shunt flow. Medium‐sized fistulas are ideally closed early to prevent further growth, as closing larger fistulas carries a heightened risk of myocardial infarction.^2^

Hyun Woo Goo's 2021 review outlined the following as contraindications^9^ for percutaneous transcatheter closure: fistulas draining near the atrioventricular annulus, extreme tortuosity, a very small patient size that complicates the procedure, as well as multiple communications and drainage sites.

## 5 CONCLUSION

During the evaluation, the patient remained asymptomatic, and imaging studies did not reveal any clear indication for surgical or catheter‐based closure. Given its rarity and potential for asymptomatic presentation, this condition can easily go unnoticed. It can have significant clinical consequences, underscoring the importance of accurate diagnosis, management, and follow‐up for patients with CAF to ensure their well‐being.

## AUTHOR CONTRIBUTIONS

**Raúl Velázquez‐Castañeda:** Conceptualization; investigation; project administration; supervision; validation; visualization. **Ilana De La Puente‐Tawil:** Conceptualization. **Sofía Molina‐Castillo:** Investigation. **Leonardo Proaño‐Bernal:** Investigation. **Mariell Alejandra Rodríguez‐Salazar:** Investigation. **Steve Masso‐Bueso:** Investigation. **Erick Alexanderson‐Rosas:** Conceptualization; formal analysis. **Vincenzo Arenas‐Fabbri:** Conceptualization; investigation.

## FUNDING INFORMATION

No funding was required nor provided for the writing of this publication.

## CONFLICT OF INTEREST STATEMENT

The authors have no conflict of interest to declare.

## ETHICS STATEMENT

No Ethics Committee approval was required for the writing of this publication.

## CONSENT

Written informed consent was obtained from the patient to publish this report in accordance with the journal's patient consent policy.

## Data Availability

The data provided in this publication may be found in patient records at our institution, “Instituto Nacional de Cardiología Ignacio Chávez”. The authors vouch for the veracity of this data.
